# The Influence of Slice Thickness, Sharpness, and Contrast Adjustments on Inferior Alveolar Canal Segmentation on Cone-Beam Computed Tomography Scans: A Retrospective Study

**DOI:** 10.3390/jpm13101518

**Published:** 2023-10-22

**Authors:** Julien Issa, Abanoub Riad, Raphael Olszewski, Marta Dyszkiewicz-Konwińska

**Affiliations:** 1 Department of Diagnostics, Poznań University of Medical Sciences, Bukowska 70, 60-812 Poznan, Poland; 2 Doctoral School, Poznań University of Medical Sciences, Bukowska 70, 60-812 Poznan, Poland; 3 Department of Public Health, Faculty of Medicine, Masaryk University, 625 00 Brno, Czech Republic; 4 Department of Oral and Maxillofacial Surgery, Justus-Liebig-University, 35392 Giessen, Germany; 5 Department of Oral and Maxilofacial Surgery, Cliniques Universitaires Saint Luc, UCLouvain, Av. Hippocrate 10, 1200 Brussels, Belgium; 6 Oral and Maxillofacial Surgery Research Lab (OMFS Lab), NMSK, Institut de Recherche Experimentale et Clinique, UCLouvain, Louvain-la-Neuve, 1348 Brussels, Belgium

**Keywords:** diagnostic imaging, X-rays, cone-beam computed tomography, mandibular canal, tomography

## Abstract

This retrospective study aims to investigate the impact of cone-beam computed tomography (CBCT) viewing parameters such as contrast, slice thickness, and sharpness on the identification of the inferior alveolar nerve (IAC). A total of 25 CBCT scans, resulting in 50 IACs, were assessed by two investigators using a three-score system (good, average, and poor) on cross-sectional images. Slice thicknesses of 0.25 mm, 0.5 mm, and 1 mm were tested, along with varying sharpness (0, 6, 8, and 10) and contrast (0, 400, 800, and 1200) settings. The results were statistically analyzed to determine the optimal slice thickness for improved visibility of IAC, followed by evaluating the influence of sharpness and contrast using the optimal thickness. The identified parameters were then validated by performing semi-automated segmentation of the IACs and structure overlapping to evaluate the mean distance. Inter-rater and intra-rater reliability were assessed using Kappa statistics, and inferential statistics used Pearson’s Chi-square test. Inter-rater and intra-rater reliability for all parameters were significant, ranging from 69% to 83%. A slice thickness of 0.25 mm showed consistently “good” visibility (80%). Sharpness values of zero and contrast values of 1200 also demonstrated high frequencies of “good” visibility. Overlap analysis resulted in an average mean distance of 0.295 mm and a standard deviation of 0.307 mm across all patients’ sides. The study revealed that a slice thickness of 0.25 mm, zero sharpness value, and higher contrast value of 1200 improved the visibility and accuracy of IAC segmentation in CBCT scans. The individual patient’s characteristics, such as anatomical variations, decreased bone density, and absence of canal walls cortication, should be considered when using these parameters.

## 1. Introduction

The inferior alveolar canal (IAC) is an anatomical structure that carries the inferior alveolar nerve and blood vessels [1]. It originates at the mandibular foramen, passes through the mandibular body, and exits at the mental foramen [1,2]. In most cases, on conventional radiographs, IAC appears as a distinct radiolucent area bordered by superior and inferior radiopaque margins [3]. To mitigate the risk of potential nerve damage, it is essential to precisely identify the location of the IAC, particularly during procedures such as dental implant placement and extraction of impacted mandibular third molars [4,5]. This becomes even more crucial in cases of ridge atrophy [6]. The segmentation/tracing of the IAC can be more challenging in certain medical conditions, such as osteoporosis [7,8]. Lower bone density may significantly impede the visibility of the canal, especially in the mental foramen area. The precise location of the mental foramen can be an anatomical challenge due to its loop, which also requires clear identification on cross sections [9]. In this specific anatomical region, the nerve exhibits a propensity to approach the midline in closer proximity than the mental foramen itself. Moreover, the lack of cortication of the canal walls may make it difficult to mark its course and detect any changes in its path, as well as its furcation and additional branches [9,10,11].

Knowledge of the tools in tomography viewing software that can facilitate the process of determining the inferior alveolar canal is essential. Currently, the guidelines only include information on what cross sections the IAC could be assessed on [9]. Still, there are no additional recommendations on which parameters may facilitate the assessment of IAC on CBCT images. The segmentation/tracing of the IAC refers to the process of digitally outlining and delineating the boundaries of the IAC on digital radiographic images.

Cone-beam computed tomography (CBCT) provides high-resolution three-dimensional (3D) digital radiographic scans, making it an important tool for diagnosis and treatment planning [12]. CBCT imaging is based on a cone-shaped X-ray beam rotating around the patient’s head, capturing multiple two-dimensional (2D) images [13]. These images are then reconstructed into 3D scans [13]. CBCT has shown satisfactory visibility of the IAC on cross-sectional images, surpassing the capabilities of conventional 2D radiographs [14,15,16].

CBCT images can be digitally modified to improve the visibility of anatomical structures. Modifications of the display settings can include slice thickness, sharpness, and contrast adjustments, among other parameters. Slice thickness refers to the thickness of each reconstructed image slice in the CBCT scan, with smaller thickness allowing for more detailed visualization but larger thickness yielding smoother scans [17,18]. Sharpness refers to the clarity and definition of the scanned structures on a CBCT scan [17]. It is influenced by factors such as detector resolution and reconstruction algorithms [17]. The contrast parameter in CBCT is used to quantify the variation in radiodensity or radiopacity among distinct anatomical features on a CBCT scan [17]. The scan contrast can be adjusted using windowing techniques [17]. Windowing defines the range of the pixel values that are visible on display, where a wider window reduces contrast, and a narrower window increases contrast [17,19].

While the expertise of the dentist is a crucial factor in IAC segmentation, optimizing CBCT settings can significantly enhance the consistency of the process. The knowledge of dentists regarding the fundamentals of dental tomography and the utilization of CBCT remains somewhat uncertain despite its widespread adoption in dentistry [20]. Hence, this retrospective study aims to bridge this gap by assessing the influence of CBCT basic view parameters, specifically contrast, slice thickness, and sharpness, on the segmentation/tracing of the IAC. This study also involves observers with varying levels of radiology training, typically representing the varying knowledge levels of a dentist using CBCT in their practice. To the best of our knowledge, this is the first retrospective study that employs this methodology. The findings from this study can provide practical insights for dentists using CBCT, in order to optimize their workflow and software settings for more accurate IAC segmentation during their clinical practice.

## 2. Materials and Methods

### 2.1. Data Acquisition

To conduct the study, CBCT scans of 25 patients (12 male and 13 female) aged 18 to 62 years were retrieved from the Poznan University of Medical Sciences database. All scans were performed between 2020 and 2021 and met the inclusion criteria outlined in Table 1.

The scans were acquired using a Cranex 3D CBCT device (Soredex, USA) with an X-ray tube voltage of 90 kV, an X-ray tube current of 10 mA, and a voxel size of 0.25 mm. The field of view (FOV) ranged from 600 × 800 mm to 1600 × 1300 mm. The 25 scans were anonymized and stored in the Digital Imaging and Communications in Medicine (DICOM) file format. Since the IAC is present bilaterally, a total of 50 IACs were evaluated.

### 2.2. Evaluation

**Figure 1:**
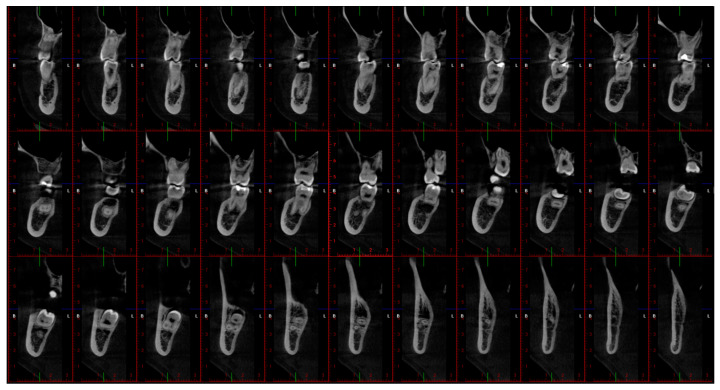
Snapshot of 33 cross-sectional images on Romexis 6.2 software (Planmeca oy, Helsinki, Finland): 0.25 mm thickness, sharpness, and contrast at zero.

Romexis 6.2 software (Planmeca oy, Helsinki, Finland) was used to process the scans and generate 33 cross-sectional images (Figure 1) from each scan for further evaluation. This software was used as it facilitates the export of the segmented structure as a Standard Triangle Language (STL) file. Two independent investigators (an oral and maxillofacial radiologist with over ten years of experience and a trainee in oral and maxillofacial radiology with three years of experience) evaluated the images on an NEC MultiSync EA245WMi-2 display screen (Sharp NEC DisplaySolutions, Tokyo, Japan) under optimal ambient lighting conditions. The evaluation was repeated twice, with a 10-day interval. The investigators rated the visibility of the IAC based on a 3-score classification (good, average, and poor), as shown in Table 2. The Brightness value in Romexis 6.2 software (Planmeca oy, Helsinki, Finland) was fixed to 1808 by default.

Slice thicknesses of 0.25 mm, 0.5 mm, and 1 mm were evaluated, with sharpness and contrast settings set to zero. The software’s default configuration has sharpness and contrast set at zero, allowing us to test four different combinations. Subsequently, a rigorous statistical analysis was executed to pinpoint the optimal slice thickness value for achieving enhanced visibility of the IAC. Following this, the investigators examined the influence of varying sharpness values (6, 8, and 10) on IAC visibility using the slice thickness value that yielded the best results. Lastly, the impact of diverse contrast values (400, 800, and 1200) was assessed using the previously identified optimal combination of slice thickness and sharpness settings.

### 2.3. Evaluation of 3D Models

After obtaining the results, the recommended image display parameters value of slice thickness, sharpness, and contrast were applied on Romexis 6.2 (Planmeca oy, Helsinki, Finland). Using the IAC tracing option in Romexis 6.2 (Planmeca oy, Helsinki, Finland), the investigators independently conducted a semi-automated segmentation of the 50 IACs. This segmentation process was executed on a cross-sectional view with a fixed cylindrical diameter of 1.5 mm. The resulting segmentation data were then saved as individual STL files and subsequently exported to Cloud Compare v.2.13.alpha (open-source software available at http://www.cloudcompare.org/ accessed on 7 April 2023) for further analysis.

Cloud Compare was employed to perform a 3D registration, enabling the overlap and visualization of the segmented IACs produced by both investigators from the same scan. The objective of this process was to evaluate the accuracy of the segmentation performed by investigators with varying levels of expertise while adhering to the recommended parameters value. This evaluation focused on assessing the level of conformity between the segmented structures by analyzing volumetric deviations, thereby evaluating the practicality and effectiveness of the recommended parameter values.

In the initial steps of this assessment, a pre-registration process was carried out using the 3-point method within Cloud Compare v.2.13.alpha (open-source software available at http://www.cloudcompare.org/ accessed on 7 April 2023. For each segmented IAC, three points were strategically placed on each of the two obtained 3D models (STL file) at corresponding locations, specifically the mandibular foramen, molar, and premolar area. This step ensured the proper alignment of the 3D models in the spatial domain.

**Figure 2:**
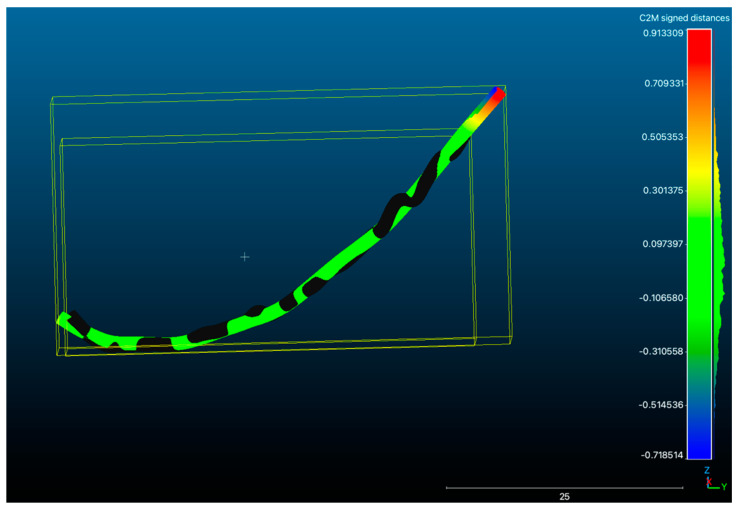
IAC overlapping and visualization of 3D comparison deviation chromatogram on Cloud Compare v.2.13.alpha (open-source software, http://www.cloudcompare.org/ accessed on 7 April 2023). The blue color represents the minus direction of deviation, the red color represents the plus direction of deviation, and the green color represents the average value.

Following this alignment process, the ‘compute cloud/mesh distance’ function in Cloud Compare v.2.13.alpha (open-source software, http://www.cloudcompare.org/ accessed on 7 April 2023 was implemented. This function overlapped the two 3D models and generated numerical results, which included parameters like the mean distance and maximum distance (Figure 2). The software default setting for the overlap parameter was set at 100, indicating that the surfaces were configured to have full overlap, equivalent to 100% overlap, in this analysis.

### 2.4. Statistical Analysis

Statistical analysis was conducted using SPSS 29.0 (SPSS Inc., Chicago, IL, USA). Inter-rater and intra-rater reliability of the IAC visibility ratings were assessed using Kappa statistics, both between the two investigators and within each investigator, across the two evaluation sessions. 

The inferential statistics were performed using Pearson’s Chi-square test with a significance level of <0.05. To evaluate the degree of conformity between the structures represented by the volumetric deviations obtained from the segmentation of the IACs, the mean distance and standard deviation were computed, and the average was calculated.

## 3. Results

Table 3 presents the results of inter-rater and intra-rater reliability analysis of the operators for slice thickness, sharpness, and contrast. The findings indicate that the operators achieved a significant level of reliability for all three parameters. The inter-rater reliability percentages for slice thickness, sharpness, and contrast are 79%, 69%, and 76%, respectively. Meanwhile, the intra-rater reliability percentages for the same parameters are 83%, 83%, and 81%, respectively.

Table 4 indicates that the visibility of IAC was most consistently rated as “good” with a slice thickness of 0.25 mm, as 80% of the 50 IACs were rated as such. This is in comparison to 70% and 66% of IACs rated as “good” with slice thicknesses of 0.5 mm and 1 mm, respectively.

Similarly, Table 4 shows that the sharpness value of zero had the highest frequency of agreement among investigators in rating IAC visibility as “good” at 80%, followed closely by a sharpness value of 10 at 78% (Table 5). Sharpness values of 6 and 8 also had a high frequency of agreement at 76% (Table 5). In Table 6, the highest frequency of agreement among investigators for good visibility of IAC was found at a contrast value of 1200, with 82% of IACs rated as “good”. A contrast value of 400 (Table 6) and 0 (Table 4) also had 80% of IACs rated as “good”, while a contrast value of 800 (Table 6) had 76% of IACs rated as “good”.

Table 7 presents the findings of the overlap analysis conducted by both investigators, focusing on the mean distance and standard deviation. The analysis was performed for each side (left and right) of the included patients. Across all patients’ sides, the average mean distance and standard deviation values were determined to be 0.295 mm and 0.307 mm, respectively.

## 4. Discussion

The interpretation of dental radiography, especially concerning the segmentation of the IAC, is notably influenced by factors such as the acquisition parameters, image quality, and experience of the dentist. While the assessment of image quality is subjective and can vary among individuals, the primary goal is to ensure that the images provide sufficient information for clinical decision making. In this retrospective study, we aimed to investigate the impact of several CBCT view parameters, contrast, slice thickness, and sharpness on the accuracy of IAC segmentation.

Regarding slice thickness, our findings revealed that a slice thickness of 0.25 mm resulted in the highest frequency of IAC visibility rated as “good” by both investigators. This suggests that thinner slices enhance the visualization and differentiation of the cortical border of the IAC from surrounding structures, while a study by Pour et al. evaluated the effect of slice thickness (0.5 mm, 1 mm, 2 mm) on the visibility of IAC in CBCT images and concluded that slice thickness has no effect on the visibility of IAC [21]. In terms of sharpness, our study demonstrated that a sharpness value of zero exhibited the highest frequencies of agreement among investigators for rating IAC visibility as “good.” This implies that a moderate level of sharpness contributes to better visualization of the IAC in CBCT scans. The evaluation of contrast values indicated that a contrast value of 1200 yielded the highest frequency of agreement among investigators for rating IAC visibility as “good.” This implies that higher contrast values enhance the visibility of the IAC in CBCT scans.

A semi-automated segmentation of the IACs was performed to validate the identified parameters, followed by an overlap analysis. The mean distance to conformity is one of the metrics that can be used to quantitatively evaluate the overlapping comparison [22]. The results showed a mean distance of 0.295 ± 0.307, indicating a reasonable level of conformity between the volumetric deviations of the segmented structures. This validation confirms the reliability and accuracy of the identified parameters in enhancing IAC segmentation, irrespective of the evaluator’s experience. Notably, the two investigators involved in the study had different levels of experience.

When applying these parameters, it is crucial to consider individual patient characteristics and specific clinical requirements. Factors such as patient age, sex, bone quality, and anatomical variations should be considered to ensure optimal image interpretation and clinical decision making. In a study by Miles et al. [23], the effect of age, gender, and location on the visibility of IAC was evaluated. The findings indicated that age had an impact on the visibility of the IAC, but this effect varied by location [23]. The first premolar region, specifically in the age range of 47–56, exhibited lower visibility compared to individuals aged over 65 [23]. Gender also played a significant role, with females generally having lower visibility than males, and the most pronounced difference was observed in the first premolar area [23].

Furthermore, other image settings such as field of view (FOV), bit depth, resolution, and the CBCT device brand should be considered. In a study by Kamburoğlu et al. [24], the Veraviewepocs 3D model X550 (J Morita Mfg. Corp., Kyoto, Japan) was found to provide the best image quality compared to the Iluma Ultra Cone-beam CT Scanner (3M Imtec, Ardmore, OK, USA), Kodak 9000 Extra-oral imaging system (Eastman Kodak Co, Rochester, NY, USA), and Vatech PanX-Duo3D_Pano/CBCT (Vatech, Seoul, Republic of Korea) [24]. Pour et al. [25] suggested that exporting mandibular CBCT images with a resolution of 0.32 mm and a 12-bit depth would yield good-to-moderate radiographic visibility of the IAC. Jasa et al. [26] conducted an in vitro study to assess the impact of exposure parameters and slice thickness on the visibility of clear and unclear IAC. The study revealed that detecting unclear IACs required higher exposure parameters or processing the images with thicker slices, whereas clear IACs could be adequately detected using lower exposure parameters [26].

In recent years, advancements in artificial intelligence (AI) have revolutionized the fields of oral and maxillofacial radiology. Numerous studies have explored the application of AI for IAC segmentation on both 2D and 3D radiographs, yielding promising results [27]. The integration of AI technology holds the potential to establish a globally standardized approach to dental reporting, providing support to dentists, streamlining their workflow, and ultimately leading to improved patient outcomes [27].

This pioneering retrospective study presents a unique approach, engaging observers of varying levels of radiology training in contrast to previous research. It demonstrates the potential utility of recommended parameters within image viewer software for achieving precise segmentation, regardless of the clinician or specialist’s expertise. Furthermore, this study introduces an innovative method that involves 3D spatial overlap of the segmented IAC for validation of recommended parameter values. This method offers promising avenues for further research in the domain of oral and maxillofacial radiology, particularly in the context of IAC segmentation. This is especially significant, considering the limited existing literature in this field. 

The presented study has a few limitations. The sample size was relatively small, which may limit the generalizability of the findings. Future studies would benefit from a more extensive and diverse patient population to further validate the identified parameters. Additionally, exploring the impact of using alternative CBCT devices or different image viewer software on the accuracy of IAC segmentation could provide valuable insights. Moreover, investigating the influence of other CBCT view parameters, such as field of view and exposure settings, could contribute to a more comprehensive understanding of their effects on IAC segmentation accuracy.

Future studies using different viewer software and exploring a range of parameters can contribute to the development of comprehensive guidelines for working with CBCT images. These guidelines can potentially assist practitioners in achieving precise evaluations, ultimately enhancing the diagnostic capabilities of CBCT in dentistry. It is worth noting that many dentists, despite utilizing CBCT in their practice, often lack comprehensive training and may not fully benefit from these advanced imaging techniques.

## 5. Conclusions

In conclusion, our study identified that thinner slice thickness (0.25 mm), zero sharpness value, and higher contrast value (1200) could enhance the visibility and accuracy of IAC segmentation in CBCT scans. However, individual patient characteristics of the bone pattern and specific clinical requirements should be considered when applying these parameters. The process of tracing IAC can be challenging and has no easily available gold standard.

Therefore, the findings from this study can serve as an initial step in establishing extended guidelines for IAC segmentation, improving the accuracy of this process on CBCT images. Further research with a larger sample size and using other software is recommended to validate and expand these findings.

## Figures and Tables

**Table 1:** Inclusion criteria.

Inclusion Criteria	Exclusion Criteria
Patients aged 18 years old and above	Patients under 18 years old
Sufficient field of view (FOV) for visualizing the entire lower jaws	Insufficient FOV for visualizing the entire lower jaws
Dentulous or partially edentulous in the molar–premolar region	Edentulous in the molar–premolar region
CBCT scans without artifacts	CBCT scans with artifacts
Patients with periapical lesion not affecting the visibility of IAC	Patients with periapical lesion affecting the visibility of IAC

**Table 2:** Three-score classification of IAC visibility.

Score	Description
Good	The cortical border of the IAC is well visible and distinguished from the surrounding structures in the 33 cross-sectional images
Average	The cortical border of the IAC is not visible and distinguished from the surrounding structures in less than 16 images (half of the images) of the 33 cross-sectional images
Poor	The cortical border of the IAC is not visible and distinguished from the surrounding structures in more than 16 images of 33 cross-sectional images

**Table 3:** The mean of inter-rater and intra-rater reliability for the evaluation of the evaluated parameters. SD, standard deviation.

	Inter-Rater Reliability		Intra-Rater Reliability	
	Mean	SD	Mean	SD
Slice thickness	0.790	0.126	0.829	0.084
Sharpness	0.687	0.103	0.834	0.105
Contrast	0.756	0.205	0.810	0.011

**Table 4:** The 0.25, 0.5, 1 mm slice thickness evaluation. Sharpness and contrast set at value 0.

Slice Thickness			Investigator 2			*p*-Value
			Good	Average	Poor	
0.25 mm	**Investigator 1**	Good	40	1	0	<0.001 *
		Average	0	8	0	
		Poor	0	0	1	
0.5 mm						
		Good	35	0	0	<0.001 *
		Average	2	12	0	
		Poor	0	0	1	
1 mm						
		Good	33	0	0	<0.001 *
		Average	3	13	0	
		Poor	0	0	1	

* Chi-square test, significance level (*p*-value) ≤ 0.05.

**Table 5:** The 6, 8, 10 sharpness value evaluation. Slice thickness set at 0.25 mm and contrast set at 0.

Sharpness			Investigator 2			*p*-Value
			Good	Average	Poor	
6	**Investigator 1**	Good	38	2	1	<0.001 *
		Average	7	0	0	
		Poor	1	0	1	
8						
		Good	38	2	1	<0.001 *
		Average	7	0	0	
		Poor	1	0	1	
10						
		Good	33	0	0	<0.001 *
		Average	3	13	0	
		Poor	0	0	1	

* Chi-square test, significance level (*p*-value) ≤ 0.05.

**Table 6:** The 400, 800, 1200 contrast value evaluation. Slice thickness set at 0.25 mm and sharpness set at value 0.

Contrast			Investigator 2			*p*-Value
			Good	Average	Poor	
400	**Investigator 1**	Good	40	0	0	<0.001 *
		Average	1	7	0	
		Poor	0	1	1	
800						
		Good	38	1	0	<0.001 *
		Average	2	6	0	
		Poor	0	0	3	
1200						
		Good	41	0	0	<0.001 *
		Average	0	7	0	
		Poor	0	0	2	

* Chi-square test, significance level (*p*-value) ≤ 0.05.

**Table 7:** Results of the overlapping analysis.

Patient	Side	Mean Distance	Standard Deviation
1	Right	0.342	0.310
	Left	0.267	0.291
2	Right	0.273	0.293
	Left	0.233	0.248
3	Right	0.268	0.267
	Left	0.223	0.243
4	Right	0.310	0.342
	Left	0.599	0.745
5	Right	0.323	0.336
	Left	0.812	0.851
6	Right	0.255	0.257
	Left	0.202	0.229
7	Right	0.225	0.240
	Left	0.174	0.209
8	Right	0.212	0.226
	Left	0.284	0.320
9	Right	0.389	0.363
	Left	0.384	0.353
10	Right	0.348	0.387
	Left	0.233	0.253
11	Right	0.198	0.235
	Left	0.235	0.245
12	Right	0.236	0.238
	Left	0.271	0.266
13	Right	0.463	0.441
	Left	0.251	0.270
14	Right	0.278	0.264
	Left	0.352	0.386
15	Right	0.313	0.305
	Left	0.266	0.251
16	Right	0.412	0.370
	Left	0.380	0.334
17	Right	0.329	0.299
	Left	0.314	0.346
18	Right	0.209	0.225
	Left	0.247	0.263
19	Right	0.291	0.251
	Left	0.284	0.253
20	Right	0.262	0.244
	Left	0.269	0.327
21	Right	0.301	0.296
	Left	0.295	0.333
22	Right	0.189	0.210
	Left	0.208	0.240
23	Right	0.174	0.201
	Left	0.224	0.241
24	Right	0.221	0.227
	Left	0.273	0.279
25	Right	0.420	0.550
	Left	0.242	0.246
Average		0.295	0.307

## Data Availability

The data presented in this study are available on request from the corresponding author.
